# Well-being of Canadian Armed Forces Veterans and Spouses of Veterans During the COVID-19 Pandemic: Protocol for a Prospective Longitudinal Survey

**DOI:** 10.2196/34984

**Published:** 2022-01-11

**Authors:** Callista A Forchuk, Anthony Nazarov, Rachel A Plouffe, Jenny J W Liu, Erisa Deda, Tri Le, Dominic Gargala, Vanessa Soares, Jesse Bourret-Gheysen, Kate St Cyr, Maede S Nouri, Fardous Hosseiny, Patrick Smith, Gabrielle Dupuis, Maya Roth, Michelle Marlborough, Rakesh Jetly, Alexandra Heber, Ruth Lanius, J Don Richardson

**Affiliations:** 1 The MacDonald Franklin Operational Stress Injury Research Centre Parkwood Institute St. Joseph's Health Care London, ON Canada; 2 Department of Psychiatry Western University London, ON Canada; 3 Department of Psychiatry and Behavioural Neurosciences McMaster University Hamilton, ON Canada; 4 St. Joseph’s Operational Stress Injury Clinic Parkwood Institute St. Joseph's Health Care London, ON Canada; 5 Department of Epidemiology Dalla Lana School of Public Health University of Toronto Toronto, ON Canada; 6 Centre of Excellence on Post-Traumatic Stress Disorder and Related Mental Health Conditions Ottawa, ON Canada; 7 The Royal’s Institute for Mental Health Research Ottawa, ON Canada; 8 Yeates School of Graduate Studies Ryerson University Toronto, ON Canada; 9 Department of Psychiatry University of Ottawa Ottawa, ON Canada; 10 Veterans Affairs Canada Ottawa, ON Canada

**Keywords:** well-being, mental health, veterans, military, survey, COVID-19, protocol, veteran, physical health, pandemic, longitudinal survey, healthcare, treatment, family support, peer support

## Abstract

**Background:**

The COVID-19 pandemic has resulted in significant changes to everyday life, including social distancing mandates, changes to health care, and a heightened risk of infection. Previous research has shown that Canadian Armed Forces (CAF) veterans are at higher risk of developing mental and physical health conditions. Veterans and their families may face unique social challenges that can compound with pandemic-related disruptions to negatively impact well-being.

**Objective:**

This study aims to longitudinally characterize the mental health of CAF veterans and spouses of CAF veterans throughout the pandemic and to understand the dynamic influences of pandemic-related stressors on psychological health over time.

**Methods:**

We employed a prospective longitudinal panel design using an online data collection platform. Study participation was open to all CAF veterans and spouses of CAF veterans residing in Canada. Participants were asked to complete a comprehensive battery of assessments representing psychological well-being, chronic pain, health care access patterns, physical environment, employment, social integration, and adjustment to pandemic-related lifestyle changes. Follow-up assessments were conducted every 3 months over an 18-month period. This study was approved by the Western University Health Sciences and Lawson Health Research Institute Research Ethics Boards.

**Results:**

Baseline data were collected between July 2020 and February 2021. There were 3 population segments that participated in the study: 1047 veterans, 366 spouses of veterans, and 125 veterans who are also spouses of veterans completed baseline data collection. As of November 2021, data collection is ongoing, with participants completing the 9- or 12-month follow-up surveys depending on their date of self-enrollment. Data collection across all time points will be complete in September 2022.

**Conclusions:**

This longitudinal survey is unique in its comprehensive assessment of domains relevant to veterans and spouses of veterans during the COVID-19 pandemic, ranging from occupational, demographic, social, mental, and physical domains, to perceptions and experiences with health care treatments and access. The results of this study will be used to inform policy for veteran and veteran family support, and to best prepare for similar emergencies should they occur in the future.

**International Registered Report Identifier (IRRID):**

DERR1-10.2196/34984

## Introduction

### Background

As of December 2021, over 1.8 million COVID-19 cases and 29,000 deaths have been reported in Canada [1]. To limit the spread of infection, the Canadian government has implemented, for example, social distancing protocols, lockdowns, school and nonessential business closures, travel restrictions, and social gathering size limits [2]. Both the virus that causes COVID-19 as well as restrictions associated with the pandemic have triggered various cascading stressors, including concerns related to contracting or spreading the virus, social isolation, changes in employment and/or financial stability, barriers to accessing supportive services, or a scarcity of these services. The widespread exposure to health threats and their multidimensional impact may lead to adverse mental health outcomes. Research conducted across countries revealed elevated rates of anxiety and depression throughout the pandemic [3,4]. More specifically, research has shown associations of symptoms of depression and anxiety with perceived threat of COVID-19 infection, social isolation, financial and occupational insecurity, and resource scarcity [5-7]. The impact of these pandemic-related stressors may be more detrimental to populations with increased vulnerability to mental illnesses, such as military veterans.

Epidemiological surveys consistently reveal that Canadian Armed Forces (CAF) veterans experience higher rates of mental and physical health conditions relative to the general Canadian population. These include posttraumatic stress disorder (PTSD), depression, and anxiety diagnoses, as well as chronic pain, arthritis, and high blood pressure [8,9]. Recent research has also found that the presence of prepandemic mental illnesses and medical conditions has been associated with greater risk of adverse mental health outcomes during the pandemic among the general population [4,10,11]. Thus, it is plausible that veterans exposed to pandemic-related stressors may be vulnerable to adverse psychological consequences. Additionally, the high rates of psychological and physical conditions in CAF veterans result in a strong reliance on physical and mental health care services relative to the general Canadian population [12,13]. Consequently, transitions and overload in health care sectors may disproportionately impose barriers to health care services and result in unmet health care needs among veterans; this may contribute to mental health concerns directly, or indirectly through worsening health conditions.

Another contributing factor to veterans’ increased vulnerability during the pandemic involves their social networks and interpersonal relationships. Veterans frequently report feelings of loneliness and dysfunctions within their interpersonal relationships (eg [12,14]). Unprecedented government-imposed restrictions on in-person gatherings may further negatively impact veterans’ recreational, occupational, and therapeutic activities. Indeed, research has underscored the associations between social isolation and elevated symptoms of depression and anxiety in the general public (eg, [15]).

Within the family context, there is evidence for existing relationship strain and disruptions between veterans and intimate partners, especially among veterans with mental illnesses [16,17]. The accumulation of stressors experienced throughout the pandemic may exacerbate psychological symptoms and amplify relationship strain for veterans and their spouses. Moreover, spouses of veterans are the most common source of unpaid caregiving to veterans [18], which can lead to significant caregiver burden and psychological distress [19,20]. Reductions in health services due to COVID-19 paired with associated exacerbations in pre-existing health conditions may result in additional responsibilities for spouses of veterans, further impacting relationship strains and psychological symptoms.

On the opposite side of the vulnerability continuum, veterans represent a unique population that has been specifically trained to demonstrate resilience in the face of adversity and extreme circumstances [21]. Despite the profile of vulnerabilities and risks, there is reason to expect that this population may adapt well compared to the general population. Research conducted in samples of veterans during the COVID-19 pandemic has revealed nonsignificant differences between veterans and the general population in levels of psychological symptoms [22,23]. However, these studies collected data at only a single time point during the pandemic, with specific samples (ie, only CAF veterans aged 55 years or older; only UK veterans) that may not generalize to all CAF veterans. Additional research is warranted to evaluate the long-term psychological impact of the pandemic and associated stressors on the mental health of veterans. This is especially important in light of theories that avoidance behaviors common to mental illnesses, including PTSD, may be temporarily validated and reinforced [24,25]. These changes may lead to transient improvements in mental health that will create additional challenges when and if society returns to a prepandemic state.

### The Current Study

This manuscript details the protocol for a longitudinal study aiming to capture the impact of the COVID-19 pandemic on the well-being of veterans and their spouses. We will assess a variety of psychological outcomes and determine how they fluctuate according to perceptions of social support, loneliness, family functioning, changes in health care delivery and access, disease status and precautions (eg, social distancing), occupational and financial insecurity, and demographics. Future publications from this study will explore the following: (1) the impact of the COVID-19 pandemic on psychological outcomes, with consideration for possible mechanisms of action such as the mediating and/or moderating roles of social support and loneliness or occupational and financial concerns on well-being, and (2) experiences, satisfaction, and psychological/well-being outcomes related to health care access and telehealth services.

## Methods

### Study Design

We employ a prospective longitudinal panel design using the secure web-based data collection platform Research Electronic Data Capture (REDCap), hosted by Lawson Health Research Institute. Participants completed questionnaires at baseline (from July 2020 to February 2021) and provided their emails for follow-up surveys to be completed every 3 months for a total of 18 months. The survey was conducted and will be reported according to the Checklist for Reporting Results of Internet E-Surveys (CHERRIES) [26]. This study was approved by the Institutional Research Ethics Boards of Western University Health Sciences (WREM #115933) on July 16, 2020, and Lawson Health Research Institute (ReDA #10001) on July 17, 2020.

### Participant Selection and Recruitment

Participants were recruited using a convenience snowball sampling strategy. Recruitment sources included a research participation recruitment platform (ParticipAid), emails distributed across professional and veteran group networks, social media advertisements, targeted media releases, and word of mouth. Recruitment channels directed prospective participants to the ParticipAid study webpage, which offered further information on the study procedures and directed eligible participants to the REDCap survey link. Eligible participants were at least 18 years of age, currently residing in Canada, and were CAF veterans or spouses (married or common-law) of CAF veterans at the time of baseline data collection. Eligibility was self-reported by participants via screening questions presented on the recruitment website, ParticipAid, and at the outset of the baseline REDCap survey.

Veteran participants' representativeness was monitored weekly throughout the recruitment period by comparing the sampling distributions against Canadian national databases on gender, age, and province of residence [27]. Recruitment efforts were adjusted accordingly to target underrepresented demographic segments (eg, through adjusting the target audiences in paid social media advertisements). No monetary compensation was provided to participants.

### Data Collection

#### Survey Design and Administration

The online survey was designed in consultation with international research teams to evaluate the cross-cultural effects of the COVID-19 pandemic on veterans and their families. A drafted version of the survey was presented to the MacDonald Franklin Operational Stress Injury Research Centre advisory council comprising CAF veterans, spouses of CAF veterans, experts in veteran and military research, health care professionals providing treatment to veterans and military members, and other stakeholders engaged in the veteran community. The advisory council provided feedback and guidance on scale selection and relevance to issues faced by CAF veterans during the pandemic.

To cater to participant availability, participants can choose between two versions of the baseline survey: a short-form version (approximately 20 minutes) or a long-form version (approximately 30 minutes; Table 1). The short-form version consists of 9 measures, which contain between 171 and 240 items collectively (depending on conditional display logic), and is presented over 9 screens/pages. The long-form version consists of 15 measures, which contain between 221 and 312 items collectively (depending on conditional display logic), and is presented over 14 screens/pages. The long-form survey can be completed immediately at baseline or be returned to within a 6-week window after beginning the baseline measures.

After baseline, an automated follow-up survey is sent every 3 months for a period of 18 months to all participants who provided an email address for contact. The follow-up survey (approximately 30 minutes) consists of 13 measures, which contain up to 293 items collectively (depending on conditional display logic), and is presented over 13 screens/pages. To reduce participant burden, all survey versions (long-form, short-form, and follow-up) use conditional display logic to hide certain questions or modules that are not relevant (eg, hiding employment demographic questions at follow-up if their employment status did not change). Only eligibility-related questions require a response; all other items can be skipped. There is an option for participants to use a return code to change their responses once submitted. The surveys are offered in English or French based on participants’ language preferences. Psychometrically validated French scales are used when available; otherwise, professional translations of the English scales, with certificates of translation provided, are used. Implied e-consent is collected at each data collection event (baseline and at each follow-up) by completion of questionnaires.

A team of 3 researchers was responsible for testing the technical aspects of the survey (eg, numeric validation, display of items and instructions, conditional display logic). Researchers tested the survey by assuming the role of hypothetical participants that systematically varied in all conditions on which conditional display logic is based (eg, “You are a Canadian Veteran **and a** spouse and you choose the short version for now but complete additional measures after. You are a “key worker” in public safety and work full-time.”). Automated processes, including emails for follow-up surveys, were systematically tested by the same researchers for all hypothetical scenarios.

**Table 1:** Data collection tools for short-form, long-form, and follow-up surveys.

Collection of assessments/domains	Short-form survey	Long-form survey	Follow-up survey
Demographics	✓	✓	✓
COVID-19 experiences/impact	✓	✓	✓
Health care access	✓	✓	✓
Social support 1 (Multidimensional Scale of Perceived Social Support)	✓	✓	✓
Social support 2 (Perceived Social Support Questionnaire)		✓	
Posttraumatic stress disorder (Posttraumatic Stress Disorder Checklist for the DSM-5)	✓	✓	✓
Depression (Patient Health Questionnaire-9)	✓	✓	✓
Anxiety (Generalized Anxiety Disorder Scale-7)	✓	✓	✓
Moral injury (Moral Injury Outcome Scale)	✓	✓	✓
Loneliness (University of California Los Angeles - Loneliness Scale)	✓	✓	✓
Alcohol consumption (Alcohol Use Disorders and Identification Test)		✓	✓
Family function (Family Assessment Device – General Functioning subscale)		✓	✓
Positive well-being (Mental Health Continuum – Short Form)		✓	✓
Chronic pain (Brief Pain Inventory – Short Form)		✓	✓
Social desirability (Marlowe-Crowne Social Desirability Scale)		✓	

#### Questionnaires

##### Demographics

Participants are asked to report their province of residence, age, gender, marital status, characteristics of area of residence (eg, city or rural area), education level, ethnicity, living arrangements (eg, whether living alone), and employment status including changes to employment during the pandemic (eg, change in work environment, hours worked, salary). Veteran participants are asked for their rank at time of release, branch of service, and length of service. Spouses of veterans are asked whether their veteran partner has a mental health condition and if yes, whether the condition occurred as a result of occupational service.

##### COVID-19 Experiences/Impact

Using items adapted from the CoRonavIruS health and Impact Survey (CRISIS) [28], participants are asked about their exposure to, infection with, and associated consequences (eg, hospitalization) of COVID-19 for self and family members. Participants are also asked about frequency of social contacts and time spent outside the home, perceived difficulties and stress related to physical distancing recommendations, changes to the quality of close relationships, concern over living situation and finances, media consumption, and an open-ended item to describe any concerns not otherwise addressed. Additional items created for this survey assess concerns relating to the pandemic (eg, on health, business, accessing essential goods), general mental health and stress of participants and their spouses, past-week COVID-19–related behaviors (eg, avoid public places, maintain social distancing), and impacts on work. Response options vary by question and are rated on 4-point, 5-point, or 6-point Likert scales, with various yes/no options for COVID-19 exposure–related questions (eg, Yes, has positive test; Yes, medical diagnosis, but no test; Yes, have had some possible symptoms, but no diagnosis by doctor; No symptoms or signs).

##### Health Care Access

Participants are asked about whether they have had difficulties accessing health care and whether they have avoided health care with yes and no options (I haven’t needed care; Yes; No; I don’t know; Prefer not to answer), with respective questions about the specific types (eg, family doctor; dental care) and domains (mental health; physical health; both) of care. For each domain of care that was avoided or difficult to access, participants are asked about their past-week distress levels on a 5-point Likert scale ranging from 0 (not at all) to 4 (extremely). Participants are asked about their usage frequency and interest in telehealth prior to the pandemic for physical and mental health care on 4-point (usage) and 3-point (interest) Likert scales, as well as their current interest in receiving telehealth services (5-point Likert scale). Participants are also asked about telehealth experiences since the pandemic in terms of whether they have accessed it (yes/no), for which domain of care (ie, mental and/or physical, with an open-ended question to specify details), respective satisfaction with the telehealth services (4-point Likert scale), and an open-ended question for participants to indicate additional feedback on their telehealth experiences. Telehealth questions were derived from a University of Missouri quality improvement survey and other research exploring the application of telehealth and telemedicine in various populations [29-32].

##### Mental Health Questionnaires and Moral Injury

Participants are asked about their symptoms of depression (Patient Health Questionnaire-9 [PHQ-9]) [33], PTSD (PTSD Checklist for the DSM-5 [PCL-5]) [34], general anxiety (Generalized Anxiety Disorder Scale-7 [GAD-7]) [35], alcohol consumption (Alcohol Use Disorders Identification Test [AUDIT]) [36], positive well-being (Mental Health Continuum-Short Form [MHC-SF]) [37], and moral injury expression using the Moral Injury Outcome Scale (MIOS) [38 and Litz, BT, unpublished data]. An additional item was added at the end of the PHQ-9, PCL-5, and GAD-7 for participants reporting the presence of at least one symptom on the respective scale to assess whether their symptoms are “directly related to the COVID-19 pandemic,” “made worse or exacerbated by the COVID-19 pandemic,” “related to traumatic events unrelated to the COVID-19 pandemic,” or “not sure.” An item was added after the AUDIT items to assess whether participants’ alcohol consumption decreased, increased, or stayed the same relative to before the pandemic.

##### Social Support and Family Function

Participants are asked about their experiences with loneliness (University of California Los Angeles - Loneliness Scale [UCLA-LS]) [39], perceived social support (Multidimensional Scale of Perceived Social Support [MSPSS] [40], Perceived Social Support Questionnaire [F-SozU] [41]), and family functioning (General Functioning subscale from the McMaster Family Assessment Device Scale [FAD-GF]) [42].

##### Physical Health Questionnaires

Participants are asked about their symptoms of chronic pain using the Brief Pain Inventory-Short Form (BPI-SF) [43].

##### Social Desirability

Social desirability is measured using the Marlowe-Crowne Social Desirability Scale (MC-SDS) [44]. This instrument assesses whether participants exhibit a tendency to respond in a socially desirable, rather than truthful, manner. This instrument was included for psychometric analysis on measures in this study that have not yet been validated (eg, MIOS); the MC-SDS will be used to assess the discriminant validity of measures.

### Data Management

Participant data are maintained in accordance with Western University and Lawson Health Research Institute institutional regulations pertaining to participant privacy and cybersecurity. Source data including electronic consent, patient identifiers (eg, email), and all questionnaire responses are stored on REDCap, which is securely housed behind an organizational firewall, and only accessible to approved investigators through an institutional login. Each participant is assigned a numerical code that is used to label and link source data. When exported from REDCap, all data are password-protected and accessible only to members of the research team. Data quality validation is performed directly through REDCap data entry forms by specifying acceptable numerical ranges, when applicable (eg, for age, and number of children living at home), and by using logic to hide questions irrelevant to subsamples (eg, telehealth acceptability only assessed among participants indicating telehealth use). Details on missing data and attrition will be discussed alongside corresponding results in future publications.

### Data Analytic Plan

#### Descriptive and Exploratory Analyses

Quantitative descriptive statistics will be used to examine sociodemographic characteristics including age, gender, ethnicity, income, education, occupation, and illness-related variables (eg, whether currently or formerly positive for COVID-19), as well as social, psychological, physical, and health care-related variables. These will include general descriptive analyses (means and standard deviations for continuous quantitative variables, relative frequencies for categorical variables), measures of internal consistency, and correlational analyses. Analyses will be performed on SPSS Statistics (version 28; IBM Corp) and RStudio (version 1.3). Qualitative, open-ended data, including participant descriptions of the impact of the COVID-19 pandemic on their health and well-being, comments about telehealth, and descriptions of morally injurious events, will be analyzed using content analysis. Data will be analyzed using NSR NVivo 11 software (QSR International). A minimum of two independent coders blinded to study hypotheses will code responses individually using both inductive and deductive approaches [45,46]. Themes will be explored, and identified and observed trends will be summarized.

#### Longitudinal Analyses

Data pertaining to psychological well-being and distress will be analyzed using latent growth modelling and mixed modelling methods to evaluate how psychological well-being and distress evolve over time. Longitudinal models will aim to identify predictors that influence individual trajectories of mental health and distress. Analyses will explore relations between mental health functioning in relation to social support and relationships, COVID-19–related variables, and health care access and satisfaction. Modelling and mixed modelling will be conducted using RStudio.

## Results

### Survey Completion and Representativeness

**Figure 1:**
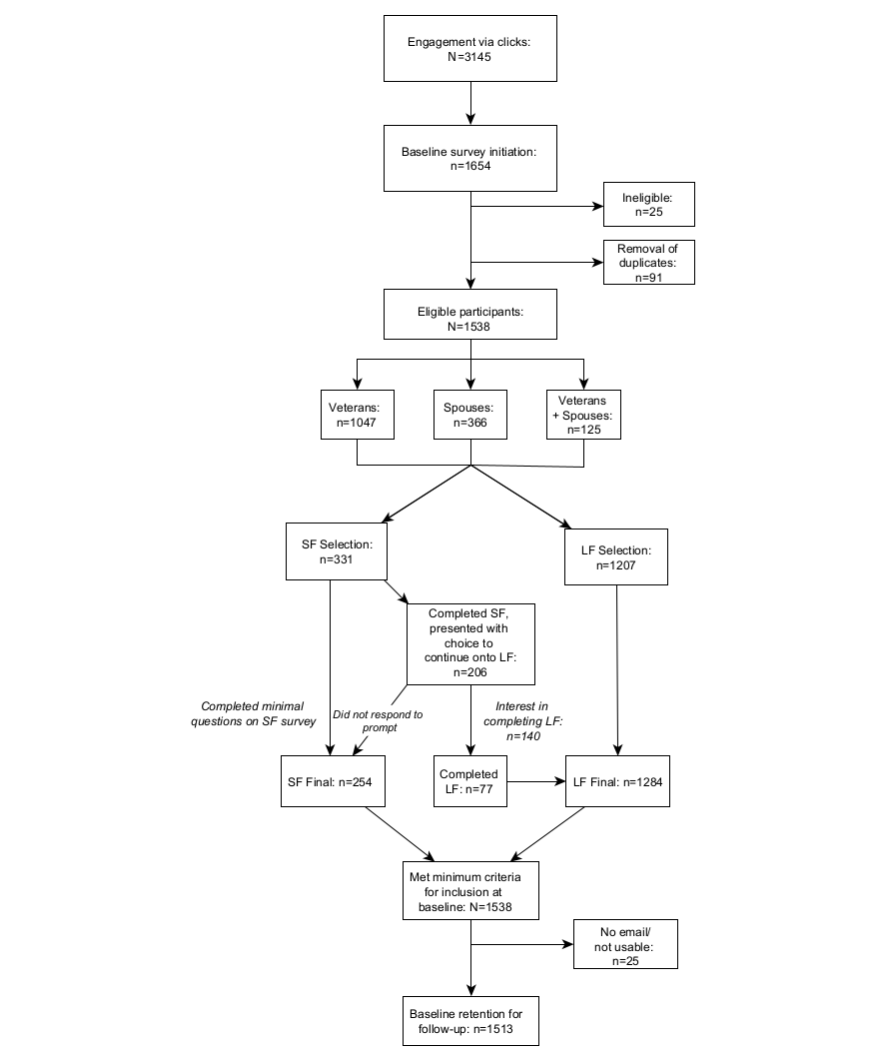
Participant flow for baseline completion rates. LF: long-form survey; SF: short-form survey.

Baseline data were collected from July 31, 2020, to February 1, 2021. A total of 1538 eligible participants initiated the survey. Among those participants, 1047 were CAF veterans, 366 were spouses of CAF veterans, and 125 were both CAF veterans and spouses of CAF veterans. Most participants who initiated the baseline survey chose to complete the long-form survey version (1207/1538, 78.5%). Most participants who completed the short-form survey (140/206, 68.0%) indicated interest in completing the additional long-form questionnaires, with 55.0% (77/140) subsequently initiating those remaining questionnaires; this represents a conversion rate of 37.4% (77/206) of those prompted, and an overall increase of 6.4% (77/1207) in long-form survey data. See Figure 1 for the flow of participants from REDCap survey initiation to baseline retention for follow-up.

The veteran participants of our baseline sample were compared against national survey data of CAF veterans [27] using chi-square goodness of fit tests on distributions of age, gender, and province of residence by Canadian regions: Atlantic Provinces, Central Canada, Prairie Provinces, West Coast, and Northern Territories [47]. Overall, our veteran sample was largely similar to the Canadian veteran population in age, gender, and region of residence. Slight deviations from the national distributions were found in each variable, with absolute percentage differences ranging from less than 1% to 11%. For age, absolute percent differences varied from less than 1% to 10%, with absolute differences greater than 5% observed in older age categories. Our sample had more participants in the 50-59 (+10%) and 60-69 (+6%) age groups, and fewer participants in the 80-89 (–10%) and ≥90 (–9%) age groups. Chi-square results revealed a significant difference in age distribution (*χ*^2^_6_=274.75, *P*<.001; Cramer V=.20, revealing this effect was small [48]). The absolute difference in proportions of males and females between our sample and the national estimate is 11.0%. Relative to national estimates, females were overrepresented in our veteran sample (*χ*^2^_1_=136.07, *P*<.001), with a medium effect size (Cramer V=.35). The absolute differences in proportions for region of residence varied from less than 1% (West Coast) up to 7% (Atlantic Provinces). The regional distribution of our participants differed from that of the national estimates (*χ*^2^_3_=44.49, *P*<.001), with a medium effect size (Cramer V=.12).

### Participant Data Retention

**Figure 2:**
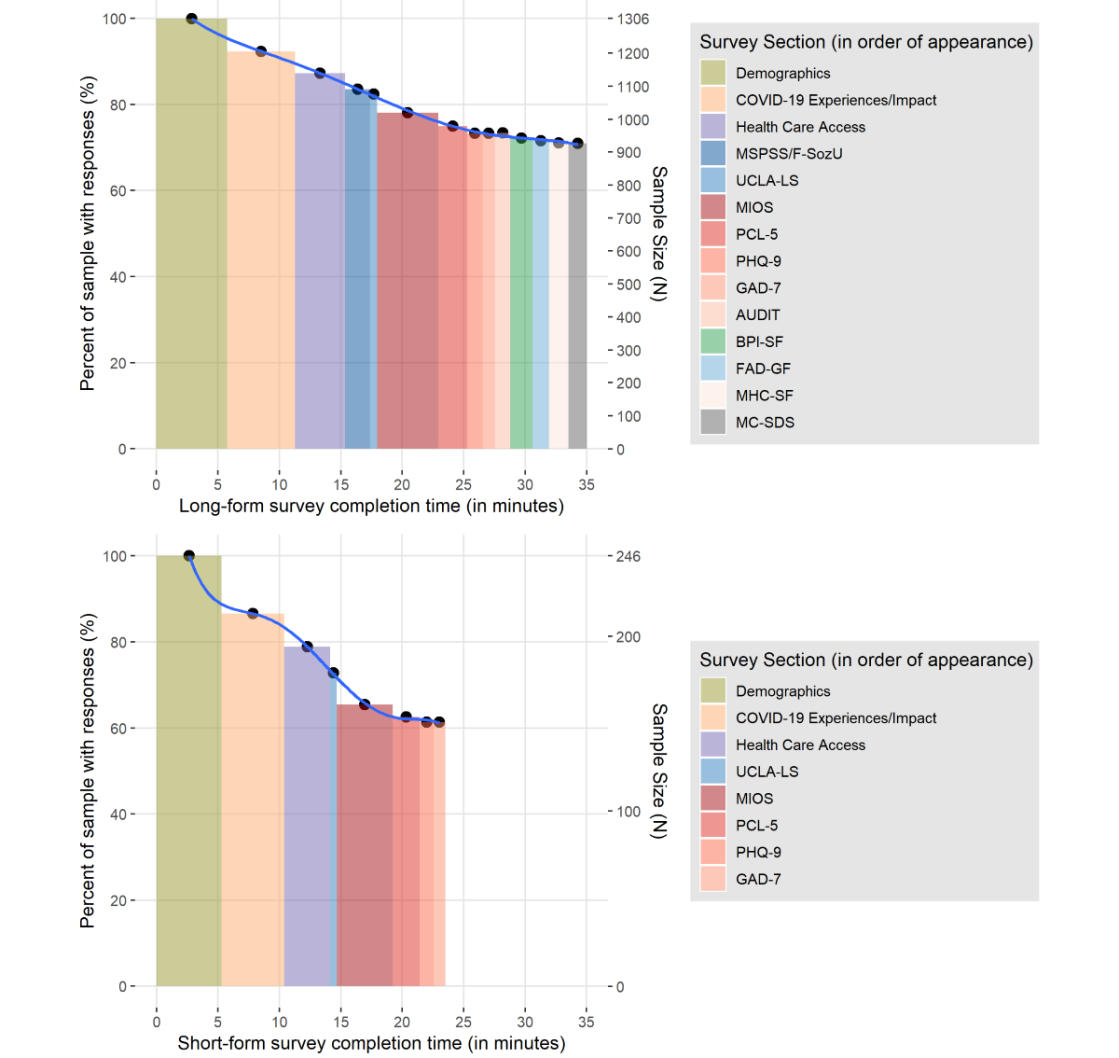
Completion rates of individual questionnaires at baseline. AUDIT: Alcohol Use Disorders and Identification Test; BPI-SF: Brief Pain Inventory – Short Form; F-SozU: Perceived Social Support Questionnaire; FAD-GF: Family Assessment Device – General Functioning subscale; GAD-7: Generalized Anxiety Disorder Scale-7; MC-SDS: Marlowe-Crowne Social Desirability Scale; MHC-SF: Mental Health Continuum – Short Form; MIOS: Moral Injury Outcome Scale; MSPSS: Multidimensional Scale of Perceived Social Support; PCL-5: Posttraumatic Stress Disorder Checklist for the DSM-5; PHQ-9: Patient Health Questionnaire-9; UCLA-LS: University of California Los Angeles - Loneliness Scale.

The median survey completion time varied by 11.5 minutes between the short-form (median 23.5 minutes) and long-form (median 35.0 minutes) versions of the survey. The percentages of participants completing each questionnaire at baseline varied according to whether participants completed the long or short version of the survey. The drop-off trend was similar between both survey lengths. For the long-form version, completion dropped from 100% at demographics to around 90% for the COVID-19 factors questions, with participation remaining above 80% for health care access, social support (MSPSS), and loneliness questions (UCLA-LS), before dropping to approximately 80% in the moral injury questionnaire (MIOS), and ranging between 70%-80% for the remainder of the questionnaires (PCL-5, PHQ-9, GAD-7, AUDIT, BPI-SF, FAD, MHC-SF, and MC-SDS). Participants who initiated the short-form version exhibited a similar trend. The rate of decline was steeper, particularly from the health care access questions to the MIOS questionnaire, with the completion rate of the full set ranging between 60% and 65% (Figure 2).

Based on the date of baseline survey initiation, participants receive system-generated notifications to complete their follow-up surveys every 3 months for a total of 18 months. The baseline retention with email addresses (N=1513) is being used as the reference point to evaluate participant retention and attrition during each of the follow-up periods. As of November 2021, a total of 831/1513 (54.9%) participants completed the 3-month follow-up, whereas 682/1513 (45.1%) missed the response window, and a total of 667/1513 (44.1%) participants completed the 6-month follow-up, and 846/1513 (55.9%) missed the response window. Data collection is ongoing into the 9-month and 12-month follow-up periods, with 683/1513 (45.1%) and 58/1513 (3.8%) participants missing the response window for each of these data collection periods, respectively. Participants missing the response window for follow-up time points will still be contacted for the next follow-up. Data collection over the full 18-month follow-up period is projected to end in mid-September 2022.

## Discussion

This paper details the protocol for a longitudinal study evaluating the impact of the COVID-19 pandemic on the well-being of Canadian veterans and spouses of Canadian veterans. The use of convenience sampling and a flexible survey format yielded a large, cross-national sample of veterans and spouses. The longitudinal data gathered as part of this study will provide key insights in mapping out the long-term consequences of the COVID-19 pandemic and associated changes on dimensions of well-being among veterans and spouses. Our findings will contribute to the identification of vulnerabilities and protective factors that may enhance or reduce well-being in veterans and their spouses throughout the pandemic, and during similar emergencies should they occur in the future. Moreover, findings from this study will contribute to the optimization of health care for veterans and their families. A strength of this study is the comprehensive and rigorous development of the online survey. Given the wide-ranging implications of the COVID-19 pandemic, it is crucial to survey an exhaustive range of dimensions to understand their multifaceted and interactive impacts on well-being. Further, this survey was developed with leading researchers and health care providers for veteran communities and was refined with feedback from veterans and spouses. The likelihood of feasibly capturing relevant experiences is high. The longitudinal design of this study is another important strength for several reasons. It is essential to capture data across several time points due to the evolving nature of the pandemic. Dynamic changes, such as fluctuating public health restrictions and regulations, waves of community outbreaks, and the progressive (and highly politicized) development of COVID-19–related research and vaccines underscore the value of longitudinal data collection. In addition, the pandemic will likely have complex indirect influences on veterans’ and spouses’ well-being through mediating roles of social support, social isolation, and/or financial insecurity. Finally, long-term monitoring is needed to capture lagged impacts of the pandemic on psychological well-being, which have been anticipated as society moves toward notions of a “new normal” [49].

There are several challenges associated with this study. These include maintaining a sizable response rate over the data collection period. Strategies were employed at the outset of data collection to achieve a large baseline sample size in anticipation of participant attrition. For example, both long-form and short-form survey designs were incorporated in this study to encourage participation among those who may have otherwise declined due to time constraints; the use of long-form and short-form survey versions has been successful in other longitudinal studies [50]. Ongoing retention strategies include sending reminder emails and tracking rates of attrition to monitor sample sizes during follow-up periods.

As of November 2021, data collection is ongoing, with participants currently completing surveys for the 9- and 12-month follow-up periods; data collection will be completed in mid-September 2022. This study offers a unique opportunity to characterize the impacts of the pandemic on veterans through both their own perspectives and through the lenses of their spouses. The results of this study will be used to inform policy and treatment planning to better support Canadian veterans and their families in the aftermath of the pandemic and during similar future events.

## Abbreviations

AUDIT: Alcohol Use Disorders and Identification Test
BPI-SF: Brief Pain Inventory – Short Form
CAF: Canadian Armed Forces
CHERRIES: Checklist for Reporting Results of Internet E-Surveys
CoE: Centre of Excellence on Post-Traumatic Stress Disorder and Related Mental Health Conditions
CRISIS: CoRonavIruS health and Impact Survey
F-SozU: Perceived Social Support Questionnaire
FAD-GF: Family Assessment Device – General Functioning subscale
GAD-7: Generalized Anxiety Disorder Scale-7
MC-SDS: Marlowe-Crowne Social Desirability Scale
MHC-SF: Mental Health Continuum – Short Form
MIOS: Moral Injury Outcome Scale
MSPSS: Multidimensional Scale of Perceived Social Support
PCL-5: Posttraumatic Stress Disorder Checklist for the DSM-5
PHQ-9: Patient Health Questionnaire-9
PTSD: posttraumatic stress disorder
REDCap: Research Electronic Data Capture
UCLA-LS: University of California Los Angeles - Loneliness Scale
